# Facile Synthesis of Mn^4+^-Activated Double Perovskite Germanate Phosphors with Near-Infrared Persistent Luminescence

**DOI:** 10.3390/nano9121759

**Published:** 2019-12-11

**Authors:** Jiaren Du, Dirk Poelman

**Affiliations:** 1 LumiLab, Department of Solid State Sciences, Ghent University, Krijgslaan 281-S1, B-9000 Ghent, Belgium; jiaren.du@ugent.be; 2 Center for Nano- and Biophotonics (NB-Photonics), Ghent University, B-9000 Ghent, Belgium

**Keywords:** near-infrared emission, persistent luminescence, double perovskite, La_2_MgGeO_6_

## Abstract

Tetravalent manganese doped phosphors are emerging as a new class of efficient near-infrared emitters for applications in a variety of areas, such as bioimaging and night-vision surveillance. Novel double perovskite-type La_2_MgGeO_6_:Mn^4+^ phosphors were successfully prepared using a microwave-assisted energy-saving solid state method. This simple technique involving the use of a microwave susceptor allows for a reduction of the preparation time compared to a conventional solid state reaction. The samples were investigated using powder X-ray diffraction, scanning electron microscopy, as well as energy-dispersive X-ray spectroscopy mapping, photoluminescence excitation/emission spectroscopy, persistent luminescence decay and temperature-dependent photoluminescence analysis. Substitution between isovalent Mn^4+^ and Ge^4+^ can be achieved without additional charge compensators in this germanate-based phosphor, which provides strong emission in the near-infrared spectral region, assigned to the characteristic transitions of tetravalent manganese ions. Additionally, the double perovskite-type germanate phosphor exhibits excellent luminescence thermal stability. Moreover, the spectroscopic properties, excitation wavelength-dependent and temperature-dependent persistent luminescence were studied. A series of thermoluminescence measurements were presented trying to give clear information on the charging process, afterglow behavior and the nature of the traps responsible for the persistent luminescence. The present investigation expands the range of available promising near-infrared emitting persistent phosphors for medical imaging.

## 1. Introduction

Persistent luminescence, also named long afterglow, is the optical phenomenon where light emission can persist for an appreciable time after the excitation source has been switched off. Materials with such self-sustained light emitting features are essential for a variety of applications in the fields of emergency lighting, anti-counterfeiting, night-vision signage, in vivo bio-imaging, and optical data storage [1,2,3,4,5,6]. The principles behind persistent luminescence of inorganic phosphors are related to traps and emitters. Emitters release light in the wavelength range of interest which usually comes from electronic transitions, such as 3d → 3d (Mn^4+^, Cr^3+^), 4f → 4f (Eu^3+^) or 5d → 4f transitions (Ce^3+^, Eu^2+^). Traps store the excitation energy and the number of available traps determine the intensity and duration of the persistent luminescence. The release rate of carriers captured in traps depends on ambient thermal energy available and the energy needed to release the carriers from the traps.

Persistent phosphors emitting in the visible spectral region have drawn extensive attention, for instance, CaS:Eu^2+^, Dy^3+^ with red emission, SrAl_2_O_4_:Eu^2+^, Dy^3+^ with green emission, Sr_2_MgSi_2_O_7_:Eu^2+^, Dy^3+^ with blue emission and CaAl_2_O_4_:Eu^2+^, Nd^3+^ having violet emission [7,8,9,10]. Development of persistent phosphors in wavelengths beyond the visible spectral region, particularly in the near-infrared (NIR), has been of great significance, ranging from night-vision surveillance to medical imaging. Biomarkers or nanoparticles with persistent luminescence in the NIR optical window can be used as ideal agents for high-sensitivity medical imaging as they allow bio-imaging without external excitation, without autofluorescence and with high signal-to-noise ratio in biological tissues [11,12]. This state-of-the-art bio-imaging technology has motivated the exploration of NIR emitting persistent phosphors with high radiance and long duration. 

Tetravalent manganese (Mn^4+^) doped phosphors have been applied as a red color component for phosphor-converted white light-emitting diodes [13]. Mn^4+^ ions with 3d^3^ electron configuration can substitute for Al^3+^, Ga^3+^, Si^4+^, Ti^4+^, Zr^4+^, and Ge^4+^ ions and be stabilized in octahedral symmetry environment of several hosts. However, in regard to NIR persistent luminescence, the number of the reported Mn^4+^ doped phosphors is very limited [14,15,16,17]. Phosphors with double perovskite crystal structure have attracted increasing attention, due to their unique properties and potential applications. The double perovskite structure with the general chemical formulation A_2_BB’O_6_, features an ordered arrangement of corner-sharing [BO_6_] and [B’O_6_] units between the two different octahedrally coordinated B and B’ cations. Cationic ordering of B and B’-site sublattices plays an important role in determining the phosphor properties. Ge^4+^ ions are ordered on the octahedral sites in the La_2_MgGeO_6_ double perovskite-type structure which provides suitable sites for Mn^4+^. The preparation of La_2_MgGeO_6_ materials usually involves a two-step thermal treatment, which requires high annealing temperatures (around 1400 °C) for a total period from 8 to 14 h [18,19,20]. To our knowledge, no alternative preparation methods for the Mn^4+^ doped La_2_MgGeO_6_ have been presented to date. In addition, some key properties regarding its persistent luminescence, such as thermal stability, charging process and wavelength/temperature-dependent afterglow behavior are investigated in detail for the first time.

In this work, double perovskite-type La_2_MgGeO_6_:Mn^4+^ phosphors were successfully prepared by a microwave-assisted solid state (MASS) method in combination with subsequent brief calcination. This facile microwave-assisted technique allows for a significant reduction of preparation time compared to the conventional solid state method. The dielectric constant (ε) and dielectric tangent loss (δ) of the materials are both important for the conversion of microwaves to heat. Despite the fact that most ceramic materials interact poorly with microwave radiation, a microwave susceptor (e.g., activated charcoal) can be employed as a heating convertor to absorb the microwave energy fully. MASS preparation is an environmentally friendly and cost-effective method. With the use of inexpensive domestic microwave ovens, it has been applied in a variety of luminescent materials for yielding products with high homogeneity and purity. The La_2_MgGeO_6_:Mn^4+^ materials show strong emission in the near-infrared spectral region, assigned to the ^2^Eg → ^4^A_2g_ transitions of tetravalent manganese ions. The structure, spectroscopic properties, thermal stability, excitation wavelength-dependent and temperature-dependent persistent luminescence were studied. Moreover, the infrared afterglow decay in absolute radiance unit of mW/sr/m^2^ was shown. A series of thermoluminescence (TL) measurements were performed, giving clear information on the charging process, afterglow behavior, and the nature of the traps responsible for the persistent luminescence.

## 2. Materials and Methods 

### 2.1. Materials Preparation

Mn-substituted La_2_MgGeO_6_ phosphors were prepared to start from stoichiometric amounts of the precursors La_2_O_3_ (Sigma Aldrich, Saint Louis, MO, USA, 99.99%), MgO (Alfa Aesar, Karlsruhe, Germany, 99.95%), GeO_2_ (Alfa Aesar, Karlsruhe, Germany, 99.999%), and MnO_2_ (Alfa Aesar, Karlsruhe, Germany, 99.997%). The substitution amount of Mn dopant was regulated as La_2_MgGeO_6_:xMn^4+^ (x = 0.25%, 0.5%, 1%, 2%, and 4%). The molar % is defined with respect to one mole of a host phosphor to make the intended composition with chemical formula La_2_MgGe_1−x_Mn_x_O_6_. 

For comparison, the samples were also prepared by conventional high temperature solid-state reaction (SSR). The appropriate stoichiometric amount of the starting powders were weighed and manually ground in an agate mortar. Subsequently, the powders were mixed with ethanol and put in a ZrO_2_ grinding jar. Grinding was performed in a Retsch PM 100 Planetary (Retsch Inc., Haan, Germany) ball mill for 6 hours to reduce the particle size. After evaporating the remaining ethanol, thorough grinding was performed to reduce particle agglomeration. Then the samples were heated using a two-step treatment employing a heating rate of 300 °C/h in a tube furnace (ETF30-50/18-S furnace, ENTECH, Ängelholm, Sweden). The initial calcination temperature was 1000 °C for 3 h, and the secondary calcination temperature was chosen from 1000 °C to 1400 °C for 8 h in air. All the prepared samples were well ground again after they were cooled down naturally to room temperature. 

For the samples prepared by the microwave-assisted solid-state (MASS) reaction, 10 g of powdered activated charcoal was used as the microwave susceptor [21]. Two alumina crucibles were used, including a bigger volume crucible (30 mL) to hold charcoal and a second one (5 mL) to place 0.4 g of the starting mixed precursors. This inner and smaller crucible was pushed into the charcoal to surround the precursors with a sufficient amount of the microwave susceptor in order to allow the reaction to proceed to completion fully. The bigger crucible was partially covered with an alumina disk to hold the reaction temperature. Subsequently, the two crucibles were placed into a cavity of a block of high temperature aluminosilicate thermal insulation bricks. Finally, the materials were irradiated in a domestic microwave oven (frequency: 2.45 GHz). The temperature profile of the microwave heating process follows a rapid temperature increase in a short period of time [22]. The power levels and duration time of irradiation were optimized and chosen as 1000 W for 40 min. These parameters were found to be highly reproducible from a number of trials. Then a thermal treatment at 1400 °C was carried out to form the final compounds with duration ranging from 1 to 3 h in air. The MASS procedure followed the previous work of Miranda de Carvalho et al. [22,23,24].

### 2.2. Characterization

Crystallographic phases of the obtained samples were verified with powder X-ray diffraction (XRD) measurements using a Siemens D5000 (Siemens, Aubrey, TX, USA) diffractometer (40 kV, 40 mA) with Cu Kα1 radiation (λ = 0.154 nm). The XRD data were collected with a 0.02° step size and 1.5 s integration step time in the range 2θ from 10° to 80° at room temperature. The measured XRD patterns were compared with the standard data from the structures of the corresponding host lattices. 

Scanning electron microscopy (SEM) was performed using a Hitachi S-3400 N (Hitachi, Berkshire, UK), equipped with a Thermo Scientific Noran System 7 for energy-dispersive X-ray spectroscopy (EDS) mapping. The samples were mounted on conductive carbon tape. SEM-EDS measurements were performed at a pressure of 25 Pa, and the EDS mappings were recorded with an accelerating voltage of 20 kV.

Steady state photoluminescence (PL), photoluminescence excitation (PLE) spectra and trap filling spectra (TFS) of the La_2_MgGeO_6_:Mn^4+^ phosphors were measured using a high resolution Edinburgh FS920 (Edinburgh Instruments Ltd., Livingston, UK) fluorescence spectrometer with a monochromated 450 W Xe-arc lamp as the excitation source. PL and PLE spectra were obtained by scanning a wavelength range from 600 to 800 nm and 240 to 550 nm respectively. 

The diffuse reflection spectra were recorded using a Perkin Elmer Lambda 1050 UV-Vis-NIR (PerkinElmer, Inc., Waltham, MA, USA) spectrophotometer with an integrating sphere, using Al_2_O_3_ as a reference. 

An integrating sphere (LabSphere GPS-SL series) was used to measure emission, absorption efficiency, the external quantum efficiency (EQE) and internal quantum efficiency (IQE) of the phosphor upon 325 nm excitation. For these measurements, white BaSO4 powder was used as a reflective standard. Absorption efficiency, IQE and EQE were calculated by using the following equations:

(1) $$\alpha_{abs}=\frac{\alpha }{\delta }=\frac{\int E_{R}-\int E_{S}}{\int E_{R}},$$

(2) $$\eta_{e}=\frac{\varepsilon }{\delta }=\frac{\int L_{S}}{\int E_{R}},$$

(3) $$\eta_{i}=\frac{\varepsilon }{\alpha }=\frac{\int L_{S}}{\int E_{R}-\int E_{S}},$$

where *ε* is the number of photons emitted by the sample, *δ* is the number of total photons excited by the light source and *α* is the number of photons absorbed by the sample. *L_S_* is the luminescence emission spectrum of the phosphor; *E_R_* is the spectrum of the excitation light with BaSO_4_ in the sphere; *E_S_* is the spectrum of the excitation light with the sample in the sphere. 

Excitation wavelength-dependent persistent luminescence profiles were obtained by monitoring at a wavelength of 709.5 nm after 3 min irradiation with variable excitation wavelength. Persistent luminescence excitation spectra or trap filling spectra were collected by integrating the persistent luminescence for 2 min. An Oxford Optistat CF cryostat with helium flow was used to investigate the temperature dependence of the optical spectra. All spectra were automatically corrected for detector response. The thermal quenching behavior of La_2_MgGeO_6_:Mn^4+^ was recorded in the range from room temperature up to 220 °C using a ProEM1600 EMCCD camera attached to an Acton SP2300 monochromator (Princeton Instruments, Trenton, NJ, USA).

Persistent luminescence decay profiles at room temperature were measured using a photosensor amplifier (Hamamatsu C9329, Hamamatsu Photonics, Hamamatsu, Japan) and a Centronics OSD100-5T silicon photodiode (Centronic Ltd., Croydon, UK). Excitation lasted for 5 min by the light of an unfiltered Xenon arc lamp at an intensity of 1000 lux. The afterglow decay profiles were then calibrated in absolute radiometric units (in mW/sr/m^2^), since photometric units are not applicable for NIR luminescence. At elevated temperatures, afterglow decay profiles were recorded by a ProEM1600 EMCCD camera.

A lab-built vacuum chamber with a well-characterized cooling and heating stage was used for a series of thermoluminescence measurements. Thin pressed pellets of samples were connected with the heat exchanger by a thermally conductive adhesive. Prior to each TL experiment, thermal cleaning of the traps was conducted. All samples were optically charged using the 254 nm emission line of a 3 W Hg-lamp for 10 min. A constant heating rate, β of 60 °C/min, was applied during the heating stage for TL measurements. Various heating rates were also tested to compare the TL profiles. The light emitted from samples during TL was guided by an optical fiber and collected using a ProEM1600 EMCCD camera connected to an Acton SP2300 monochromator. TL glow curves were plotted versus temperature in the range from 0 °C to 220 °C by integrating the TL emission spectra from 650 nm to 800 nm. Emission spectra were also collected during the charging stage, and the same spectrum range from 650 nm to 800 nm was integrated. The integrated intensity was plotted as a function of charging duration. TL measurements at temperatures in the range from 0 to 225 °C could be performed in a reliable way and the maximum 225 °C was fixed, and no higher temperature was applied in order to restrain the thermal quenching (TQ) effects.

## 3. Results and Discussion

### 3.1. Synthesis and Structural Characterization

Figure S1 presents the crystal structure of La_2_MgGeO_6_ viewed along the axis on the basis of the Inorganic Crystal Structure Database (ICSD No. 97016). The compound crystallizes in a hexagonal cell with space group R3H (space group number 146) and lattice parameters a = b = 5.5125 Å, c = 13.3295 Å, volume = 350.79 Å^3^ and z = 3 [20]. La_2_MgGeO_6_ belongs to the double perovskite-type (A_2_BB’O_6_) structure group in which Mg^2+^ and Ge^4+^ ions are ordered on B and B’ sites with six oxygen ions forming [MgO_6_] and [GeO_6_] octahedrons. Notably, the La^3+^ ion is at the center of 12 nearest neighbor O^2−^ ions forming a polyhedron. Generally, there are three types of units: [LaO_12_] polyhedrons, [MgO_6_] and [GeO_6_] octahedrons. The two neighboring octahedral units [MgO_6_] and [GeO_6_] are arranged alternately, sharing corner with one oxygen atom. This spatial arrangement of units in the La_2_MgGeO_6_ double perovskite-type structure increases the distance between adjacent luminescent centers, improving the efficient NIR luminescence from the ^2^E_g_ → ^4^A_2g_ emission process of Mn^4+^ ions. Taking into account the same valence state, charge equilibrium substitution, and the same ionic radius, as shown in Table 1, Mn^4+^ dopants preferably occupy the octahedral Ge^4+^ sites. In addition, divalent manganese (Mn^2+^) ions which are often found accompanying Mn^4+^ ions, could also occupy the six-coordinated sites, due to their similar ionic radius compared with Mg^2+^ and Ge^4+^ ions. 

The conventional solid state reaction at high temperature has been used for the synthesis of La_2_MgGeO_6_ phosphors and the effective doping of Mn in its tetravalent state. However, a long period of reaction time at high temperature involving repeated thermal treatments is usually indispensable to reduce undesired byproducts. Figure S2 presents XRD patterns of a series of La_2_MgGeO_6_ samples prepared at variable temperatures using the SSR method (initial 3 h calcinations at 1000 °C and secondary 8 h annealing at 1000 °C, 1100 °C, 1200 °C, 1300 °C and 1400 °C, respectively). The XRD patterns of these five samples along with standard XRD reference of the pure La_2_MgGeO_6_ crystal structure (ICSD No. 97016) are compared. Noticeable traces of other impurity phases were observed from samples prepared at 1000 °C and 1100 °C. These impurity phases mainly come from La_2_O_3_ precursor residuals and other intermediate phases, such as La_2_GeO_5_ (see Figure S3). At increasing calcination temperatures, up to 1400 °C, traces of impurity phases vanish gradually indicating the fact that it is essential to employ high temperature and long reaction duration in order to reduce other impurities and obtain a La_2_MgGeO_6_ single phase via the SSR method.

MASS method has been applied to reduce the time and energy consumption of the luminescent material synthesis, mainly in phosphors using a reduced state of the activators, such as Eu^2+^, Pr^3+^, Tb^3+^, and Ce^3+^-doped compounds [21,23,26]. However, this route is not directly applicable for synthesizing Mn^4+^-activated compounds because the reduction gas, usually carbon monoxide, could prevent the complete oxidation of Mn and lead to the unintended occurrence of Mn^2+^, thus, resulting in less effective doping of Mn^4+^ and the loss of NIR luminescence efficiency. Interestingly, it could be applicable for the synthesis of La_2_MgGeO_6_:Mn^4+^ by the MASS method in combination with a subsequent (mild) calcination step to achieve a trade-off: Effective doping of Mn^4+^ versus acceptable time and energy consumption. To demonstrate this, we employed a combined approach for the preparation of La_2_MgGeO_6_:Mn^4+^. The preparation was carried out simply by a 40-min MASS and a 3-h thermal treatment without intermediate grinding, which yielded the pure product of La_2_MgGeO_6_:Mn^4+^. A comparison of the XRD patterns of MASS and SSR samples with different reaction duration is shown in Figure S4. Prolonging the duration of the SSR reaction from 1h to 3 h at 1400 °C, a slight decrease of the intensity of diffraction peaks from other impurity phases was observed. Despite a prior 6-h ball mill grinding process, insufficient reactions still occur at very high annealing temperature in a relatively short period (within 3 h) for the SSR method. In addition, a 40-min MASS treatment was added before each SSR synthesis without ball mill grinding. We found that the diffraction peaks from impurities decrease substantially compared with the patterns without MASS treatment. The sample synthesized by a 40-min MASS in combination with subsequent 3-h calcination is comparable with the materials prepared by the conventional SSR method with a two-step thermal treatment for 11 h (initial 3-h heating and 8-h calcination). This observation confirmed the advantages of the MASS method employed in this work. From economical time-cost aspects, this hybrid microwave-assisted synthesis reduces the preparation time required for SSR method by more than 75%. Greatly increased productivity may come from several aspects:

(i) The reduced size of reactants. It is well established that products with reduced size are usually obtained in a variety of inorganic compounds by the MASS method [27]. It is easier to achieve complete reactions with smaller particle sizes, higher surface areas and more efficient blending of reactants.
(ii) Efficient thermal energy transfer. More homogeneous heat distribution is provided by direct heating of the samples under microwave irradiation. Moreover, the charcoal, applied as a susceptor, also contributes to heat the reactants because susceptor agents with high dielectric loss tangents usually convert microwave irradiation into thermal energy efficiently.
(iii) The preliminary formation of the host matrices. After microwave irradiation for 40 min, the crystal lattice of La_2_MgGeO_6_ was gradually formed (Figure S5), which is beneficial to reduce the total synthesis time. An additional round of 40-min MASS synthesis was tried, but the contribution to the formation of the host matrices was limited (as shown in Figure S5).

**Figure 1:**
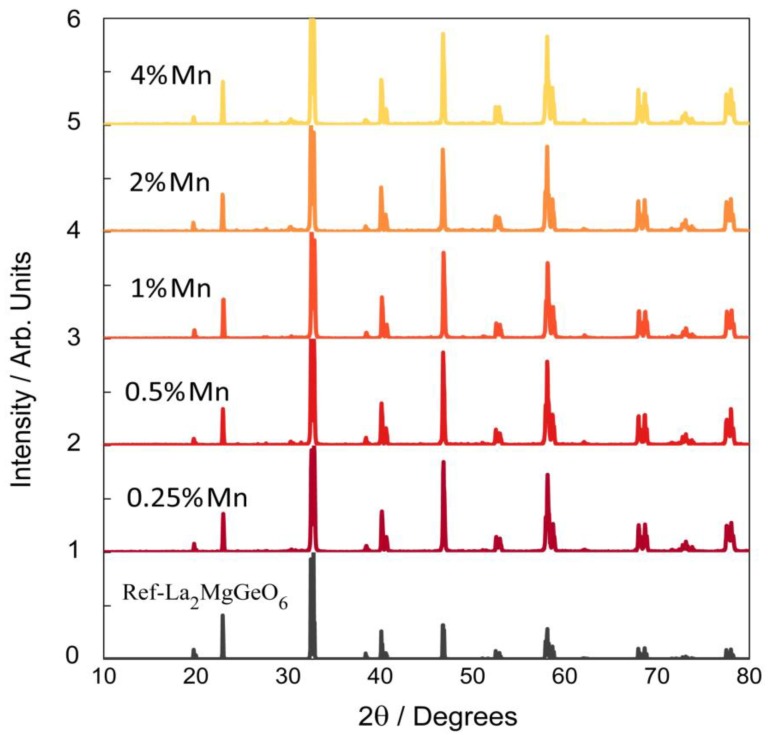
XRD patterns of La_2_MgGeO_6_:Mn^4+^ phosphors prepared using the microwave-assisted solid state (MASS) method, with variable contents of Mn dopants.

La_2_MgGeO_6_:Mn^4+^ samples with various Mn^4+^ concentrations were synthesized through this facile microwave-assisted method. The contents of Mn^4+^ dopants were 0.25, 0.5, 1, 2 and 4 molar %, respectively. The XRD patterns of these samples, along with the standard XRD reference are compared in Figure 1. All the diffraction peaks of La_2_MgGeO_6_:Mn^4+^ samples with various Mn^4+^ concentrations can be indexed to the double perovskite-type structure of the pure La_2_MgGeO_6_ phase (ICSD No. 97016), and no traces of La_2_O_3_ residual or other impurity phases were observed. The scanning electron microscope image shows the morphology of the 1 molar % sample in Figure S6, where powders are primarily on the micrometer scale. A homogeneous elemental distribution of La, Mg, Ge, O and Mn is presented, and no clusters of Mn are found.

### 3.2. Spectroscopic Properties

**Figure 2:**
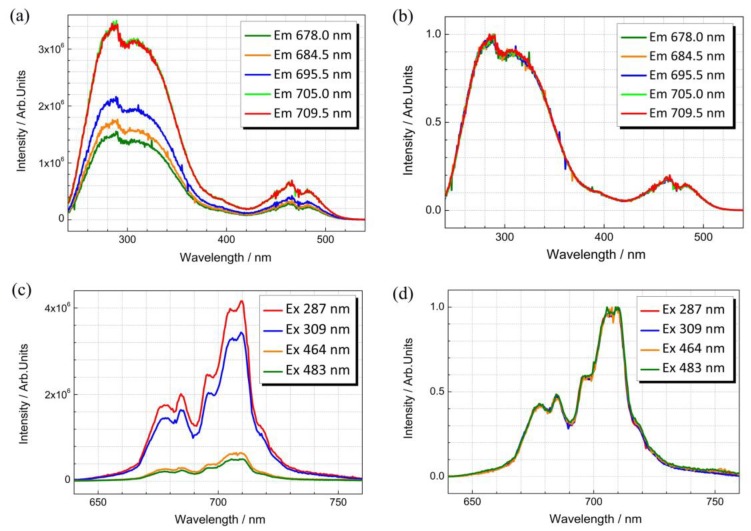
Excitation and emission spectra of La_2_MgGeO_6_:1%Mn^4+^ at room temperature. (**a**) Excitation spectra were monitored at 678, 684.5, 695.5, 705 and 709.5 nm, respectively; (**b**) The normalized excitation spectra; (**c**) Emission spectra excited at 287, 309, 464 and 483 nm, respectively; (**d**) The normalized emission spectra.

Figure 2 displays the room temperature excitation and emission spectra of La_2_MgGeO_6_:1%Mn^4+^ synthesized using the MASS method. When monitored at variable emission wavelengths (at 678, 684.5, 695.5, 705 and 709.5 nm), there are four intense excitation bands of La_2_MgGeO_6_:Mn^4+^ centered at about 287, 309, 464 and 483 nm in the range of 240–540 nm. Monitoring the different emission peaks, the intensity of excitation bands differs, while the relative position of these excitation bands is unchanged, as shown in Figure 2a,b. In view of the intensity differences, the strong 287 and 309 nm bands are attributed to the charge transfer of Mn^4+^-O^2−^ and ^4^A_2g_ → ^4^T_1g_ transition of Mn^4+^, whereas the weak 464 and 483 nm bands are attributed to the spin-allowed ^4^A_2g_ → ^4^T_2g_ transition. Emission spectra were recorded at room temperature with the excitation bands at 287, 309, 464 and 483 nm, respectively. Upon ultraviolet or blue light excitation, several emission bands are present, centered approximately at 678, 684.5, 695.5, 705 and 709.5 nm, as displayed in Figure 2c. These sharp peaks are assigned to the typical ^2^E_g_ → ^4^A_2g_ transition together with Stokes and anti-Stokes phonon sidebands of Mn^4+^ ions in an octahedral environment. The strongest NIR emission peak at 709.5 nm can be assigned to the Stokes vibronic band, which is very similar to the peak value of 14,104 cm^−1^ (circa 709 nm) reported elsewhere [19]. According to the spectroscopic properties measured at low temperature by Srivastava et al., the zero-phonon line occurs at 14535 cm^−1^ (c.a. 688 nm) and the Stokes vibronic bands at longer wavelength side are attributed to the ungerade modes of the [MnO_6_] octahedral moiety [19]. The spin-forbidden ^2^E_g_ → ^4^A_2g_ transition is partially allowed resulting from the electron-phonon coupling in the La_2_MgGeO_6_ host. The emission lines at shorter wavelength side of the zero-phonon line can be assigned to the anti-Stokes phonon sidebands. Irrespective of the excitation wavelength (see Figure 2d, the normalized emission spectra are all coincident, indicating identical emission transitions from Mn^4+^ ions. The above-mentioned spectroscopic properties suggest the incorporation of Mn ion in its 4+ oxidation state, since Mn^2+^ displays totally different broadband spectral features [28]. These spectroscopic features of the La_2_MgGeO_6_:Mn^4+^ phosphors are quite similar in samples synthesized by both MASS and SSR method (see the spectroscopic comparison in Figure S7). 

Excitation and emission spectra of La_2_MgGeO_6_:Mn^4+^ samples prepared by MASS method with various Mn^4+^ concentrations are illustrated in Figures S8 and S9. Monitoring at the same emission wavelength of 709.5 nm, the shapes of the excitation spectra of La_2_MgGeO_6_:Mn^4+^ samples with different Mn^4+^ contents are consistent (in Figure S8). Similarly, upon the same excitation at 309 nm, emission spectra show no shift in the peak position with increasing Mn^4+^ content (Figure S9). Furthermore, both the photoluminescence emission and excitation intensity initially increase with increasing of the concentration of Mn^4+^ from 0.25% to 1%, and then decrease gradually with further increasing Mn^4+^ concentration. High concentrations of dopants usually reduce the average distance of dopants and enhance the interaction among the activator centers, resulting in concentration quenching with lower luminescence efficiency. To further evaluate the luminescence properties of different samples, a comparison of the external (η_e_), internal (η_i_) quantum efficiencies (QEs) and absorption efficiency (α_abs_) of the samples with different concentrations of Mn^4+^ doping was made. Figures S10–S13 give a comparison of emission intensity, absorption efficiency, internal quantum efficiency (IQE) and external quantum efficiency (EQE) of La_2_MgGeO_6_:x%Mn^4+^ samples with different concentrations of Mn^4+^ (x = 0, 0.25, 0.5, 1, 2, 4) by using the integrating sphere. Increasing the content of Mn^4+^ doping, the amount of light absorption is also on the increase. Considering emission intensity, internal quantum efficiency and external quantum efficiency of La_2_MgGeO_6_:x%Mn^4+^ samples, the optimal concentration of Mn^4+^ is 1%, which is also consistent with the relative PL intensity in Figure S9. Furthermore, the diffuse reflection measurements were performed on 1%Mn^4+^ doped La_2_MgGeO_6_ sample and undoped La_2_MgGeO_6_ host (in Figure S14). It clearly shows the two typical Mn^4+^ absorption bands. The absorption band covering 450 nm to 480 nm was attributed to the spin-allowed Mn^4+^: ^4^A_2g_ → ^4^T_2g_ transition, while that occurred from 310 nm supposedly originated from the Mn^4+^: ^4^A_2g_ → ^4^T_1g_ transition.

**Figure 3:**
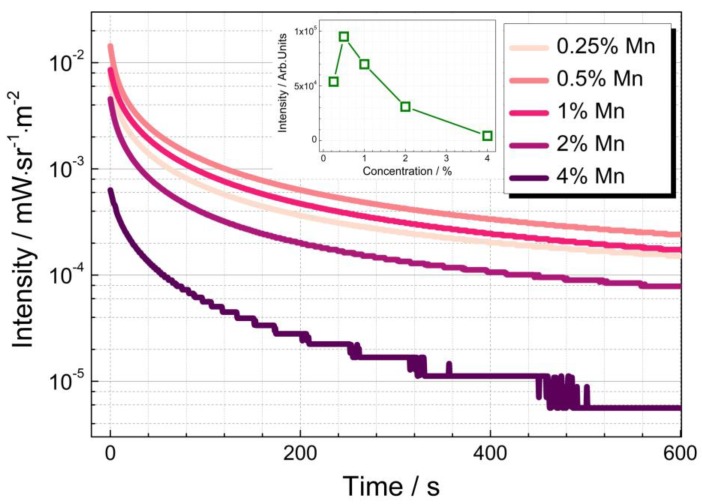
Semi-logarithmic plot of the emission decay profiles of La_2_MgGeO_6_:x%Mn^4+^ samples from 0 s to 600 s after 5 min irradiation. The inset illustrates the total afterglow intensity integrated over 10 min as a function of Mn^4+^ concentration.

To investigate the dopant concentration dependence of the persistent luminescence, the afterglow decay profiles of La_2_MgGeO_6_:x%Mn^4+^ samples (x = 0.25, 0.5, 1, 2, 4) prepared by MASS method were measured at room temperature. Figure 3 exhibits the afterglow decay curves collected from 0 to 600 s after the excitation has finished. All decay profiles exhibit fast decay rates at the first stage then decrease slowly. The afterglow decay profiles show dopant concentration dependency. When the Mn^4+^ concentration increases to 4%, there is an inferior afterglow behavior, due to the concentration quenching and the formation of defect clusters [18]. As the inset shows, the optimal Mn^4+^ content for the best afterglow performance is 0.5%. Compared with the sample doped with 0.5%Mn^4+^ synthesized by SSR method, the afterglow performance of La_2_MgGeO_6_:0.5%Mn^4+^ prepared by MASS method is better, as shown in Figure S15. It suggests the superior properties of persistent phosphors prepared using the MASS method. The samples prepared by MASS method were investigated further below. The emission wavelength-dependent afterglow decay was recorded on the sample of La_2_MgGeO_6_:0.5%Mn^4+^. Under the 254 nm excitation for 5 min, the afterglow decay was monitored at wavelength of 678, 684.5, 695.5, 705 and 709.5 nm, respectively. A logarithmic plot of the decay curves at different emission wavelengths exhibits the same linear shape in a very similar persistent luminescence behavior (in Figure S16). 

### 3.3. Luminescence Thermal Stability: Thermal Quenching

**Figure 4:**
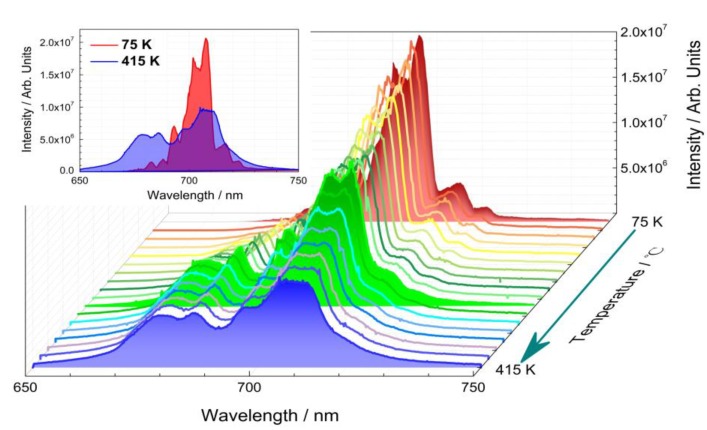
Temperature-dependent emission spectra of La_2_MgGeO_6_:1%Mn^4+^ phosphor under 309 nm excitation in the temperature range from 75 K to 415 K with a temperature interval of 20 K. Emission spectra at 75 K (in red), 275 K (in green) and 415 K (in blue) are highlighted, and the inset shows emission spectra at 75 K and 415 K.

**Figure 5:**
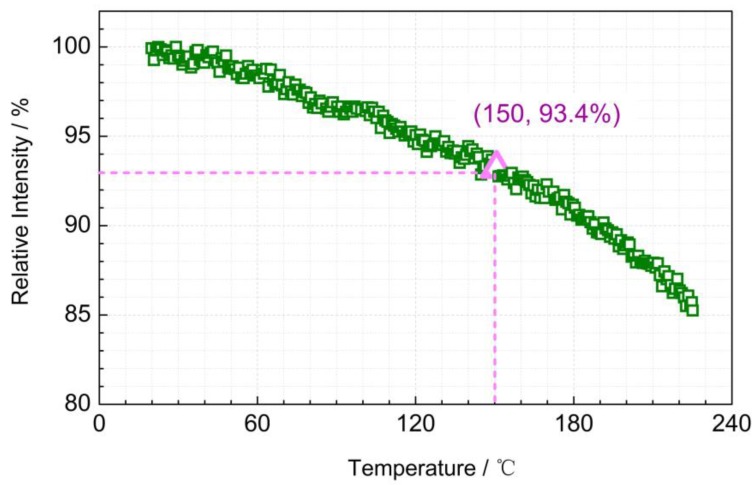
Thermal quenching behavior of the La_2_MgGeO_6_:1%Mn^4+^ phosphor up to 220 °C. The normalized integrated intensity was obtained by integrating the emission spectrum ranging from 650 nm to 800 nm.

Luminescence thermal stability of phosphors plays an important role in their practical application. Accordingly, temperature-dependent emission spectra of La_2_MgGeO_6_:1%Mn^4+^ phosphor was recorded in the temperature range from 75 K to 415 K with a temperature interval of 20 K (in Figure 4). The emission at each temperature was tested to be the steady-state photoluminescence, and the contribution from afterglow or thermoluminescence was negligible. Upon 309 nm excitation, the La_2_MgGeO_6_:1%Mn^4+^ phosphor exhibits NIR emission in the range from 650 to 750 nm. At low temperature, the emission spectrum shows the clear sharp-line featured characteristics of Mn^4+^ emission, which is composed of the zero-phonon line together with the Stokes vibronic bands and the anti-Stokes bands, in accordance to the observation reported by Brik et al. [19]. As the temperature increases, the sharp peaks decrease, and broad luminescence bands can be observed in Figure 4. This is mainly due to the increase of thermal vibrations and the rise of the anti-Stokes emissions with increasing temperature: Thermal vibrations contribute to the spectrum broadening, and the anti-Stokes contribution increases. Consequently, the emitted photons are distributed over a wider range of wavelengths, and the peak intensities decrease, even if the total intensity remains almost the same. The thermal quenching behavior up to 220 °C for La_2_MgGeO_6_:1%Mn^4+^ phosphor is shown in Figure 5. The normalized total intensity was obtained by integrating the emission spectrum of the La_2_MgGeO_6_:1%Mn^4+^ phosphor ranging from 650 nm to 800 nm, showing excellent thermal stability. Compared with the initial intensity at 20 °C, 93.4% of the total intensity remains at 150 °C. Even at 220 °C, the integrated emission intensity is still about 85% of that at 20 °C. This limited luminescence thermal quenching behavior of La_2_MgGeO_6_:Mn^4+^ phosphor makes it much superior to other Mn^4+^ doped persistent phosphors reported [29]. 

### 3.4. Excitation Wavelength-Dependent Persistent Luminescence

**Figure 6:**
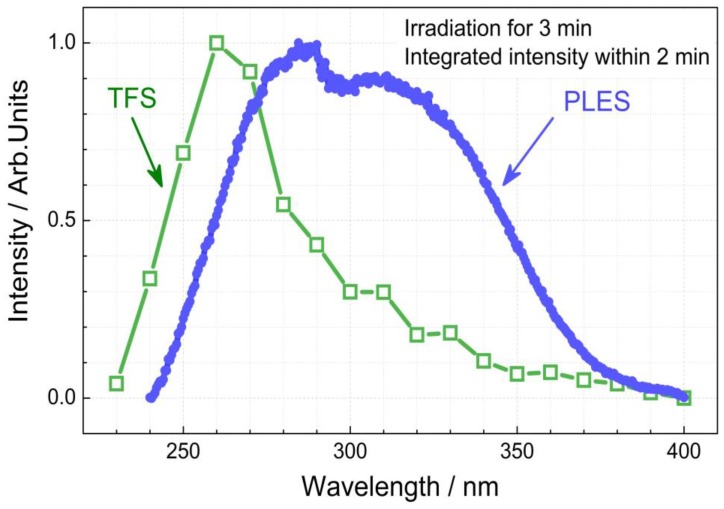
Comparison of trap filling spectrum (TFS) and photoluminescence excitation spectrum (PLES) of the La_2_MgGeO_6_:0.5%Mn^4+^ phosphor. Both PLES and TFS were monitored at 709.5 nm. TFS was obtained by integrating the persistent luminescence within 2 min after 3 min irradiation with variable excitation wavelength from 230 to 400 nm.

Furthermore, the excitation wavelength-dependent persistent luminescence was investigated to study the persistent luminescence properties of La_2_MgGeO_6_:Mn^4+^ phosphor. Although the steady-state photoluminescence in the NIR spectral region can be achieved upon excitation at a wide range of wavelengths, for instance, 309 and 464 nm, the persistent luminescence can only be effectively excited by higher photon energy light. In our experiments, the La_2_MgGeO_6_:0.5%Mn^4+^ sample was irradiated for 3 min with monochromatic light from 230 to 400 nm with a step interval of 10 nm. The excitation source was a fluorescence spectrometer with a monochromated 450 W Xenon arc lamp, and afterglow decay curves were subsequently recorded by monitoring at 709.5 nm emission. The persistent luminescence excitation spectrum or trap filling spectrum (TFS) was obtained by plotting the total integrated afterglow intensity over 2 min as a function of excitation wavelength, as shown in Figure 6. The photoluminescence excitation spectrum (PLES) of the La_2_MgGeO_6_:0.5%Mn^4+^ phosphor is displayed as well in Figure 6 for comparison. Both PLES and TFS were monitored at a wavelength of 709.5 nm. Figure 6 clearly shows that the NIR persistent luminescence of La_2_MgGeO_6_:0.5%Mn^4+^ can only be effectively achieved by excitation at shorter wavelengths. This excitation wavelength-dependent persistent luminescence property is also observed in some other Cr^3+^, Ni^2+^, Pr^3+^ or Mn^2+^ activated persistent phosphors [30,31]. The excitability of the persistent luminescence from high energy light in La_2_MgGeO_6_:Mn^4+^ phosphor may result from host lattice associated defects. As Qiu et al. pointed out, the persistent luminescence is closely related to the intrinsic defects of the La_2_MgGeO_6_ host instead of Mn^4+^ associated defects, due to the fact that excitation wavelength from the d → d transitions of Mn^4+^ can only yield photoluminescence, but not persistent luminescence [18]. Recently, Pan et al. proposed a two-photo up-conversion charging concept using low-energy, high-intensity excitation sources to explain the photoluminescence excitation and persistent luminescence excitation behavior [31]. The discussion on excitation wavelength-dependent persistent luminescence behavior is still open.

### 3.5. Thermoluminescence Behavior

**Figure 7:**
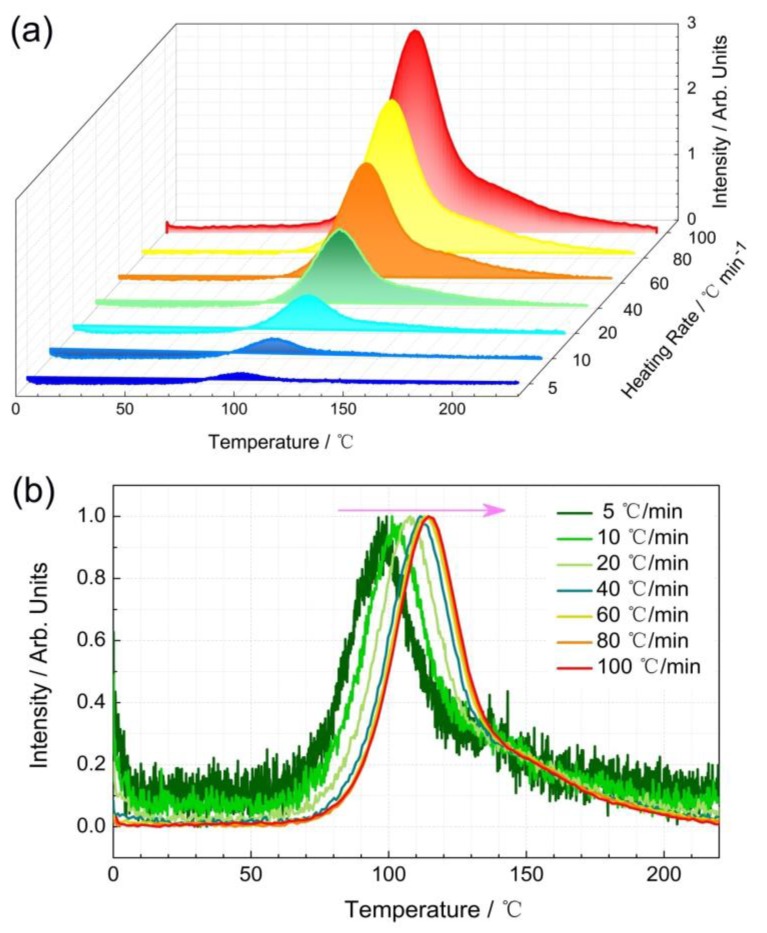
Thermoluminescence (TL) experiments with variable heating rates. (**a**) Effect of the heating rate on TL curves. The sample was optically charged using a 254 nm UV lamp for 10 min and the heating rate was chosen from 5 to 100 °C/min; (**b**) The normalized TL curves with variable heating rates as a function of temperature.

Persistent luminescence is closely associated with trap properties. To further understand the possible trapping and detrapping process, thermoluminescence was measured. A series of TL experiments with variable heating rates were performed. The La_2_MgGeO_6_:0.5%Mn^4+^ sample was optically charged using the 254 nm emission line of a 3 W Hg-lamp for 10 min. A constant heating rate β (°C/min), was applied during the heating stage for TL measurements, and the heating rate was chosen from 5 to 100 °C/min. Figure 7a clearly shows the effects of the heating rates on TL curves. Despite the differences among intensities of TL peaks, the shape of TL curves remains. The broad TL peaks originate from a continuous and broad trap distribution with different trap depth. The shape of the main TL peak is very similar to other reported glow profiles, indicating the intrinsic nature of traps in this phosphor [18]. As can be seen from the normalized TL curves in Figure 7b, the relative position of the main TL peak is shifting to the higher temperature side with increasing heating rate. The position of the TL peak maximum (T_m_, in °C) can be used to estimate the energy of trap depth (E_trap_, in eV). It is worth mentioning that in order to obtain and compare E_trap_ for each persistent phosphor, the heating rate should be well considered. Usually, a higher heating rate is chosen to improve the signal-to-noise ratio (SNR). In this way, the emitted TL light is collected in a relatively short time frame [32]. However, the risk of temperature gradients over the samples should be considered carefully when performed with a higher heating rate. Therefore, a moderate heating rate of 60 °C/min was applied for further TL measurements.

**Figure 8:**
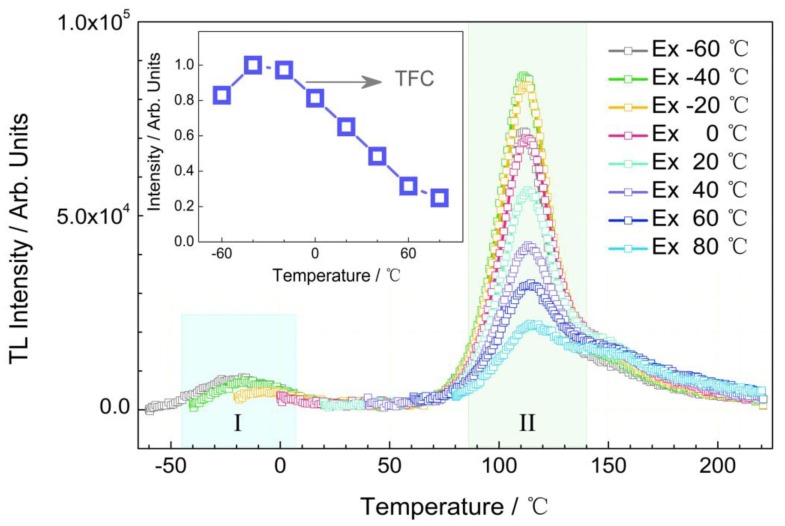
Effect of charging temperatures on TL curves. Inset shows trap filling capacity (TFC) of La_2_MgGeO_6_:Mn^4+^ phosphor as a function of charging temperature.

The effect of charging temperature on the TL curves is displayed in Figure 8. The charging temperature was set in the range from −60 °C to 80 °C with a temperature interval of 20 °C. When charged at low temperature (e.g., −60 °C), the TL curve consists of the two peak regions centered around −20 °C (region I) and 115 °C (region II). With increasing charging temperatures, the same TL peak centered around 115 °C is observed. If T_m_ represents the temperature for which the glow curve reaches a maximum, the related trap depth (E_trap_) is approximately evaluated by using the following equation:

(4) $$E_{trap}\left(eV\right)=\frac{T_{m}\left(K\right)}{500}.$$

The estimated trap depth of La_2_MgGeO_6_:0.5%Mn^4+^ phosphor is about 0.78 eV. The traps corresponding to region II, exhibiting a large amount of intensity, play a more important role in persistent luminescence than those in region I. The trap is too shallow in the region I, which makes little contribution to the RT afterglow. Thus, only traps within region II were taken into account for trap filling capacity (TFC) of La_2_MgGeO_6_:Mn^4+^ phosphor. The TFC is plotted as a function of charging temperature in Figure 8. It is interesting to note that there is a very slight thermal barrier for trap filling in the La_2_MgGeO_6_:Mn^4+^ phosphor, only seen at the very low charging temperature of −60 °C. This effective trap filling property at variable temperatures allows the use of the phosphor in different charging ambient temperatures. 

**Figure 9:**
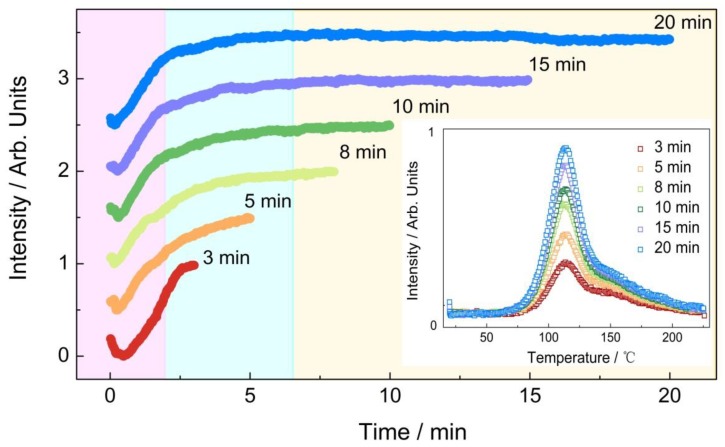
The charging process of La_2_MgGeO_6_:Mn^4+^ phosphor. The different traces are displaced vertically for clarity. The inset illustrates the TL curves with different charging time.

To further study the trapping and detrapping process in La_2_MgGeO_6_:Mn^4+^ phosphor, the charging process was illustrated in Figure 9. The total intensity of emitted light was collected from both charging stage and heating stage. The intensity was plotted by integrating from 650 to 800 nm as a function of charging time. The charging duration lasted for 3, 5, 8, 10, 15, and up to 20 min at RT, respectively. The light emission from La_2_MgGeO_6_:Mn^4+^ sample comes from (1) the steady-state PL process with relative stable intensity upon high power excitation and (2) afterglow from the persistent phosphor. The charging process of La_2_MgGeO_6_:Mn^4+^ sample can be roughly divided into three stages: In the first 2-min charging stage, the total intensity of light emission increases quickly, indicating the fast detrapping process with a large detrapping rate (afterglow). The drops of intensity observed in the first seconds are mainly due to the delayed start of the charging source. Both the trapping and detrapping process occur, and the light emission from the detrapping process is increasing within the first 2-min charging stage; subsequently, the increase of the emission intensity is going on slowly in the second stage within 5 min; ultimately, a balanced state is achieved with longer charging time, for instance, 10 min, resulting in a relatively stable emission intensity. In the latter stage, the balanced state may mainly come from the stable and constant rate of trapping and detrapping. TL measurements were performed with different charging durations. As it is shown in the inset of Figure 9, the intensity of TL peak is increasing with longer charging duration. Despite the achieved balanced state of the detrapping process, the trap filling in the region II is still on-going with longer charging duration.

### 3.6. Temperature-Dependent Persistent Luminescence

**Figure 10:**
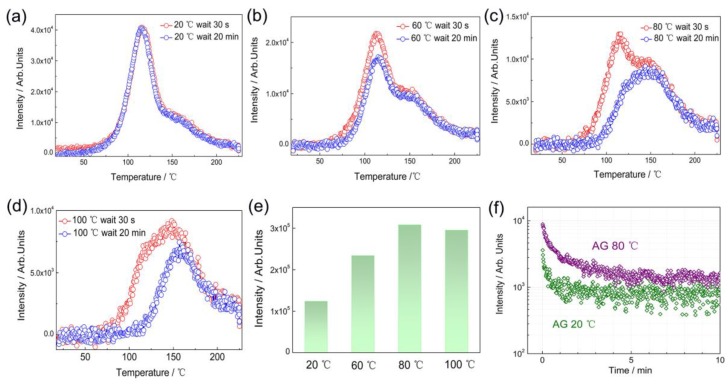
Temperature-dependent persistent luminescence of La_2_MgGeO_6_:Mn^4+^. After 10 min UV excitation, the heating stage starts after 30 s or 20 min fading time, with a constant heating rate of 60 °C/min. The charging and fading temperature were chosen at 20 °C (**a**); 60 °C (**b**); 80 °C (**c**); and 100 °C (**d**); (**e**) Comparison of integrated TL intensity difference with fading time between 30 s and 20 min, as a function of temperature; (**f**) Comparison of afterglow decay profiles at 20 °C and 80 °C.

The NIR persistent luminescence from Mn^4+^ doped phosphors (La_2_MgGeO_6_:Mn^4+^ in this work, LaAlO_3_:Mn^4+^ or GdAlO_3_:Mn^4+^) [15,16,29] is still inferior to other Cr^3+^ doped persistent phosphors reported (for instance, LiGa_5_O_8_:Cr^3+^, ZnGa_2_O_4_:Cr^3+^ or Zn_3_Ga_2_Ge_2_O_10_:Cr^3+^) [2,33,34,35]. The persistent luminescence from Mn^4+^ doped La_2_MgGeO_6_ is largely restricted to the fact that the traps are relatively deep, and thus, do not contribute significantly to the RT afterglow performance. It is known that the ideal working temperature of persistent luminescence is dependent on its trap depth and trap distribution [36]. To investigate the temperature-dependent persistent luminescence, the ambient temperature for the charging and afterglow process was chosen at 20 °C, 60 °C, 80 °C and 100 °C, respectively (in Figure 10a–d. After 10 min UV excitation, the heating stage starts at 30 s or 20 min (TL fading time) with a constant heating rate of 60 °C/min. The interval time of 20 min is sufficient to emit most of the afterglow available at that temperature. The difference obtained from the two TL fading curves (30 s and 20 min) are, therefore, proportional to the total afterglow output. Comparison of the integrated TL intensity from the two TL fading curves clearly gives a temperature-dependent afterglow behavior with an ideal afterglow temperature at 80 °C. The more stored energy can be released at an elevated temperature of 80 °C and the afterglow output at 80 °C is obviously higher than that at 20 °C, as shown in Figure 10e–f. This elevated temperature favored afterglow behavior is also observed in other Mn^4+^ phosphors [29]. 

## 4. Conclusions

In summary, a novel class of double perovskite-type La_2_MgGeO_6_:Mn^4+^ phosphors were successfully prepared by a facile MASS method. This simple microwave-assisted technique greatly reduces the preparation time compared to the conventional solid state method. Isovalent substitution between Mn^4+^ and Ge^4+^ can be achieved without additional charge compensators in this germanate-based double perovskite-type phosphor. Strong emission in the near-infrared spectral region coming from the transitions of tetravalent manganese ions can be observed. The charging process, afterglow behavior, thermoluminescence properties and the nature of the traps are studied. The strong near-infrared emitting persistent luminescence and good thermal stability make it promising for bio-imaging application. The present investigation expands the exploration of more promising near-infrared emitting persistent phosphors with different structures for medical imaging.

## Figures and Tables

**Table 1:** Effective ionic radii of the cations in La_2_MgGeO_6_ lattice [25].

Element	Valence State	Coordination Number (CN)	Ionic Radius (Å)
Mn	4+	VI	0.53
Mn	2+	VI	0.67
La	3+	XII	1.36
Mg	2+	VI	0.72
Ge	4+	VI	0.53

## Supplementary Materials

The following are available online at https://www.mdpi.com/2079-4991/9/12/1759/s1, Figure S1: Crystal structure of La_2_MgGeO_6_. The Ge^4+^ and Mg^2+^ ions are located in octahedral units, La^3+^ ions are situated in twelve-coordinated units. Figure S2: XRD patterns of La_2_MgGeO_6_ samples prepared at variable temperatures ranging from 1000 °C to 1400 °C. A comparison of the XRD patterns was made with the reference pattern La_2_MgGeO_6_ (ICSD No. 97016). Figure S3: XRD patterns of La_2_MgGeO_6_ samples and other impurity phases. The standard XRD data of La_2_O_3_ (No. 01-074-2430) and La_2_GeO_5_ (No. 00-040-1183) are illustrated. Figure S4: XRD patterns of La_2_MgGeO_6_ samples prepared by both SSR and MASS methods. Figure S5: XRD patterns of La_2_MgGeO_6_ samples prepared by MASS method with variable reaction time. Figure S6: Representative SEM image and SEM-EDS mappings in Mn^4+^-activated La_2_MgGeO_6_. Figure S7: A comparison of the normalized photoluminescence excitation and emission spectra of La_2_MgGeO_6_:0.5%Mn^4+^ samples prepared by MASS and SSR method. Photoluminescence spectra were acquired under the same excitation at wavelength of 309 nm. Photoluminescence excitation spectra were all monitored at the same emission wavelength of 709.5 nm. Figure S8: Excitation spectra of La_2_MgGeO_6_:x%Mn^4+^ samples with different concentrations of Mn^4+^ (x = 0.25, 0.5, 1, 2 and 4, respectively). Excitation spectra were all monitored at the same emission wavelength of 709.5 nm. Figure S9: Emission spectra of La_2_MgGeO_6_:x%Mn^4+^ samples with different concentrations of Mn^4+^ (x = 0.25, 0.5, 1, 2 and 4, respectively). Emission spectra were measured under the same excitation wavelength of 309 nm. The inset illustrates the total emission intensity as a function of Mn^4+^ concentration. Figure S10: A comparison of emission intensity of La_2_MgGeO_6_:x%Mn^4+^ samples with different concentrations of Mn^4+^ (x = 0, 0.25, 0.5, 1, 2, 4) by using the integrating sphere. Figure S11: A comparison of absorption efficiency of La_2_MgGeO_6_:x%Mn^4+^ samples with different concentrations of Mn^4+^ (x = 0, 0.25, 0.5, 1, 2, 4). Figure S12: A comparison of internal quantum efficiency (IQE) of La_2_MgGeO_6_:x%Mn^4+^ samples with different concentrations of Mn^4+^ (x = 0, 0.25, 0.5, 1, 2, 4). Figure S13: A comparison of external quantum efficiency (EQE) of La_2_MgGeO_6_:x%Mn^4+^ samples with different concentrations of Mn^4+^ (x = 0, 0.25, 0.5, 1, 2, 4). Figure S14: (a) Diffuse reflection spectra of the undoped La_2_MgGeO_6_ host, La_2_MgGeO_6_:1 %Mn^4+^ phosphor. (b) The ratio of the reflectance of 1% Mn doped La_2_MgGeO_6_ phosphor to the undoped La_2_MgGeO_6_ host. Figure S15: A comparison of the persistent luminescence decay profiles of La_2_MgGeO_6_:0.5%Mn^4+^ samples prepared by MASS and SSR method. The samples irradiated during 5-min prior to the decay measurement. Figure S16: The emission wavelength-dependent afterglow decay profiles of La_2_MgGeO_6_:0.5%Mn^4+^. The afterglow decay curves were recorded monitoring at 678, 684.5, 695.5, 705 and 709.5 nm, respectively.
