# Supplementary material for: Facile Synthesis of Mn4+-Activated Double Perovskite Germanate Phosphors with Near-Infrared Persistent Luminescence
Source: Nanomaterials (Basel). 2019 Dec 11;9(12):1759. doi: 10.3390/nano9121759 (PMC6955683; doi:10.3390/nano9121759)
Supplement: Supplementary file 1 [file nanomaterials-09-01759-s001.pdf]

Supporting Information

Facile Synthesis of Mn^{4+}-Activated Double Perovskite Germanate Phosphors with Near-Infrared Persistent Luminescence

Jiaren Du [1,2], Dirk Poelman [1,2*]

[1] LumiLab, Department of Solid State Sciences, Ghent University, Krijgslaan 281-S1, B-9000 Ghent, Belgium;

[2] Center for Nano- and Biophotonics (NB-Photonics), Ghent University, B-9000 Ghent, Belgium.

*Correspondence: dirk.poelman@ugent.be

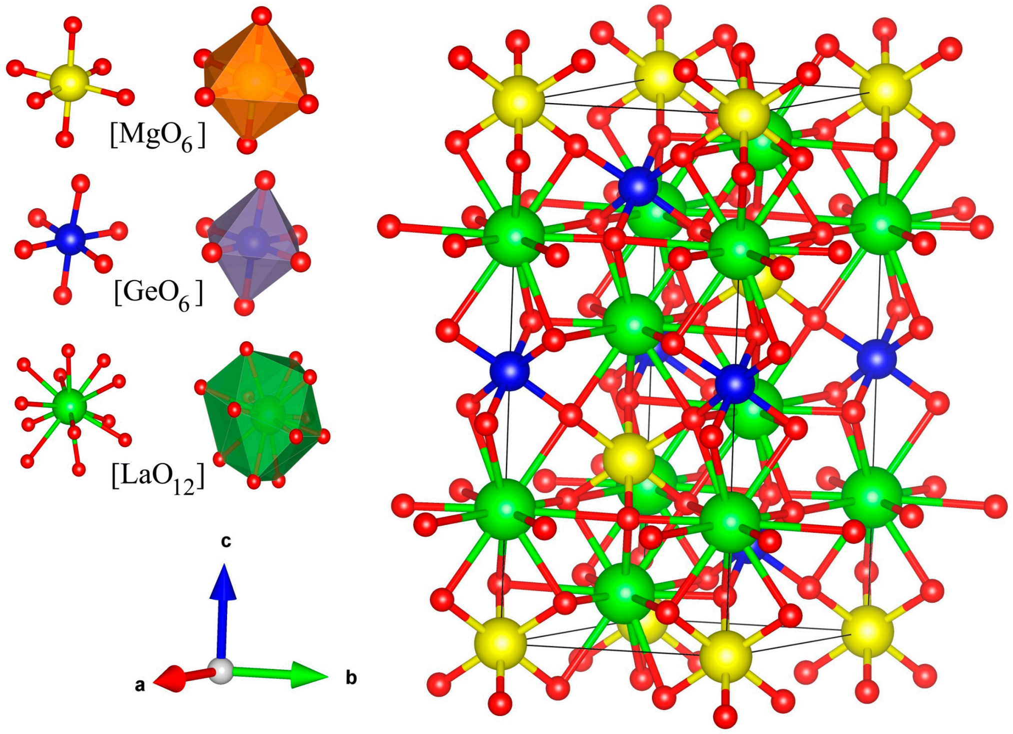

Figure S1. Crystal structure of La_2MgGeO_6. The Ge^{4+} and Mg^{2+} ions are located in octahedral units, La^{3+} ions are situated in twelve-coordinated units.

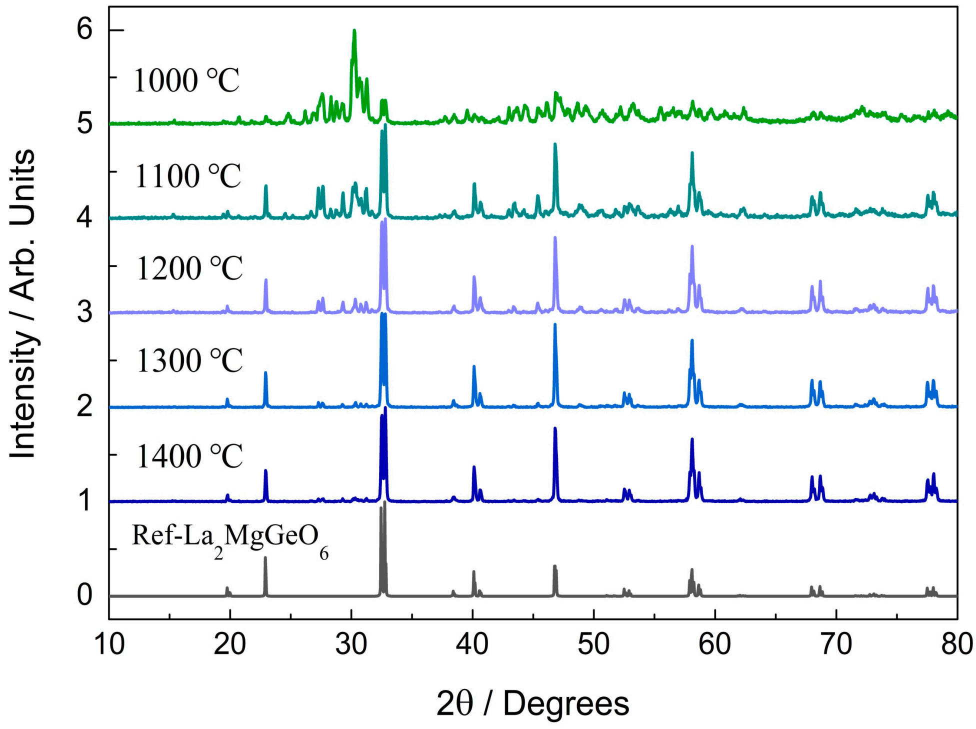

Figure S2. XRD patterns of La_2MgGeO_6 samples prepared at variable temperatures ranging from 1000 ℃ to 1400 ℃. A comparison of the XRD patterns was made with the reference pattern La_2MgGeO_6 (ICSD No. 97016).

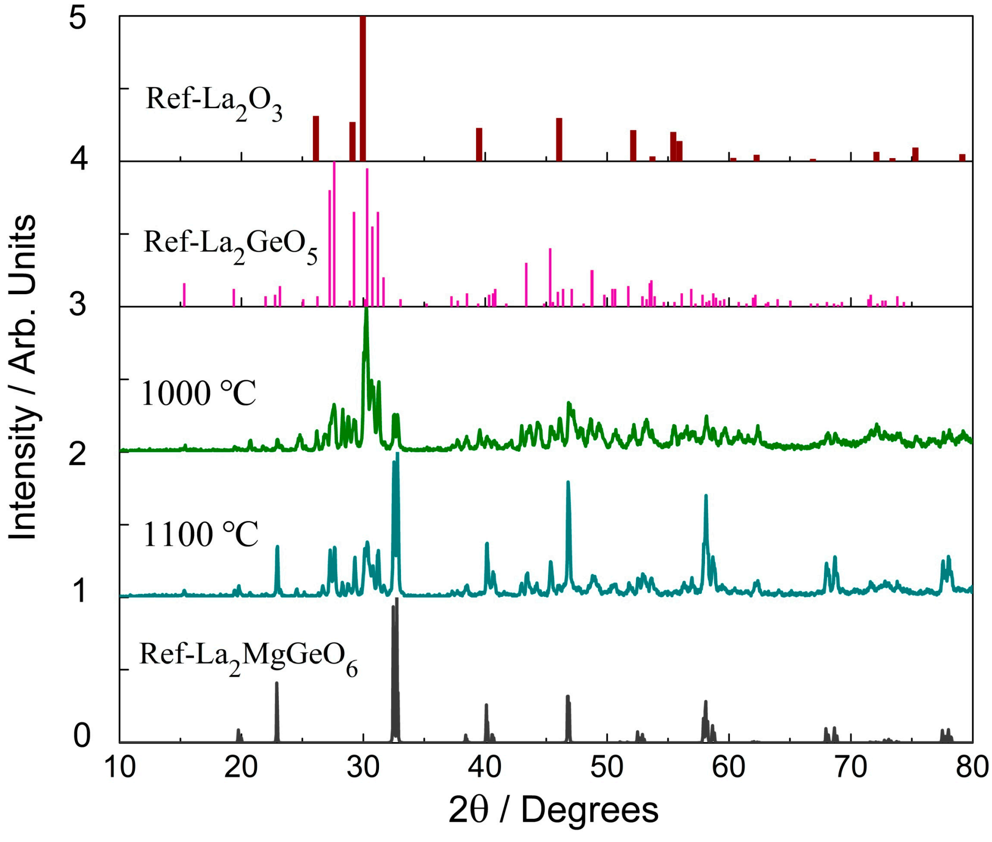

Figure S3. XRD patterns of La_2MgGeO_6 samples and other impurity phases. The standard XRD data of La_2O_3 (No. 01-074-2430) and La_2GeO_5 (No. 00-040-1183) are illustrated.

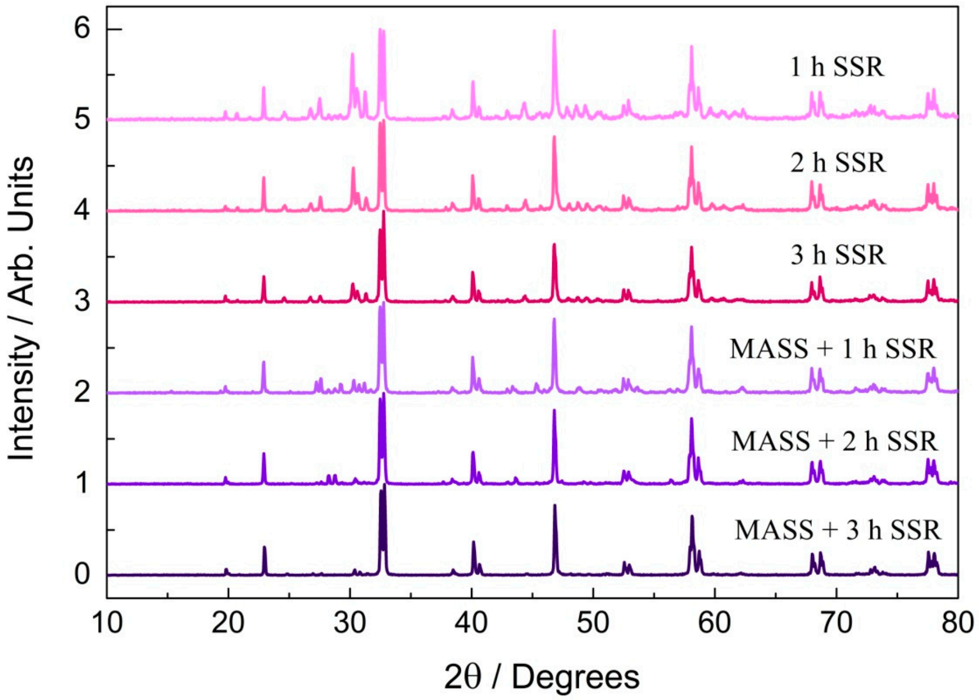

Figure S4. XRD patterns of La_2MgGeO_6 samples prepared by both SSR and MASS methods.

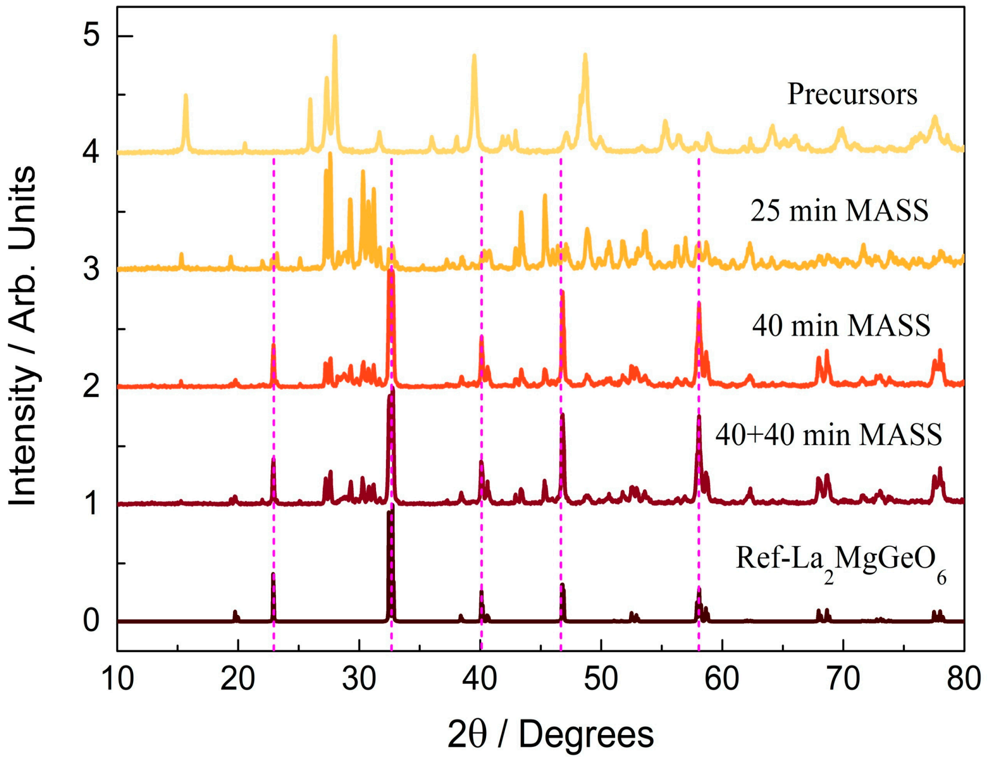

Figure S5. XRD patterns of La_2MgGeO_6 samples prepared by MASS method with variable reaction time.

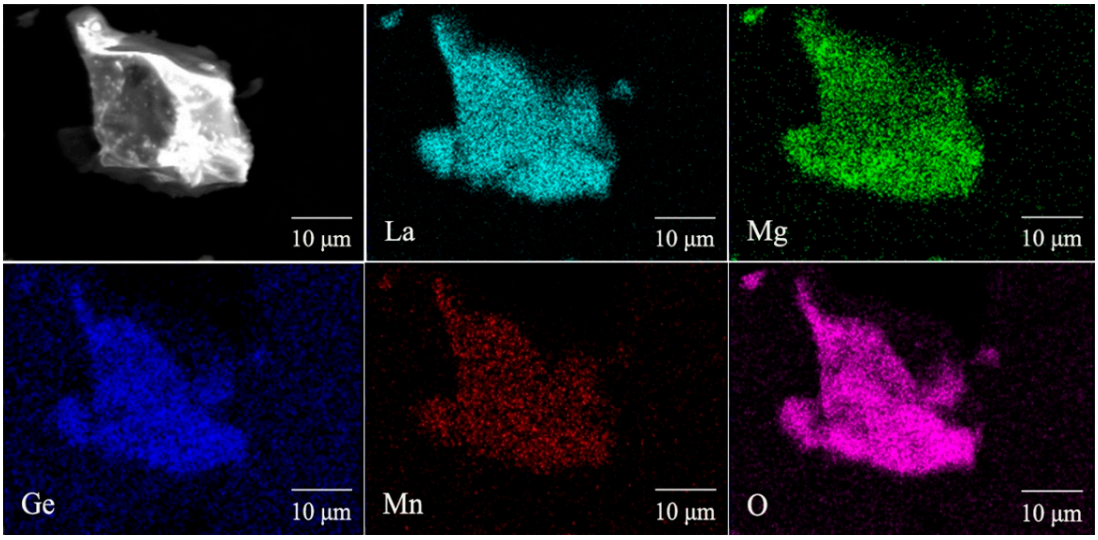

Figure S6. Representative SEM image and SEM-EDS mappings in Mn^{4+}-activated La_2MgGeO_6.

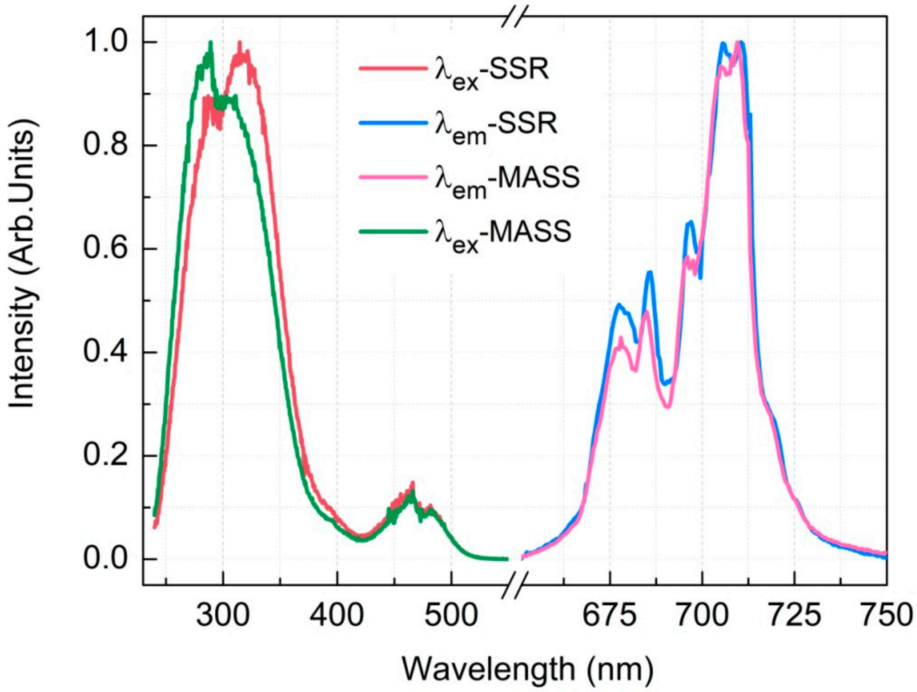

Figure S7. A comparison of the normalized photoluminescence excitation and emission spectra of La_2MgGeO_6:0.5%Mn^{4+} samples prepared by MASS and SSR method. Photoluminescence spectra were acquired under the same excitation at wavelength of 309 nm. Photoluminescence excitation spectra were all monitored at the same emission wavelength of 709.5 nm.

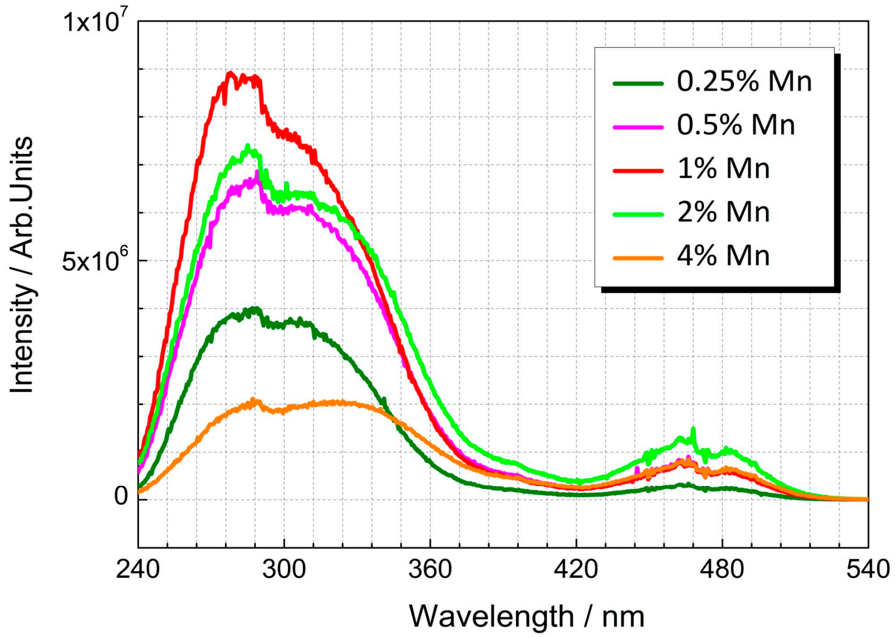

Figure S8. Excitation spectra of La_2MgGeO_6:x%Mn^{4+} samples with different concentrations of Mn^{4+}(x = 0.25, 0.5, 1, 2 and 4, respectively). Excitation spectra were all monitored at the same emission wavelength of 709.5 nm.

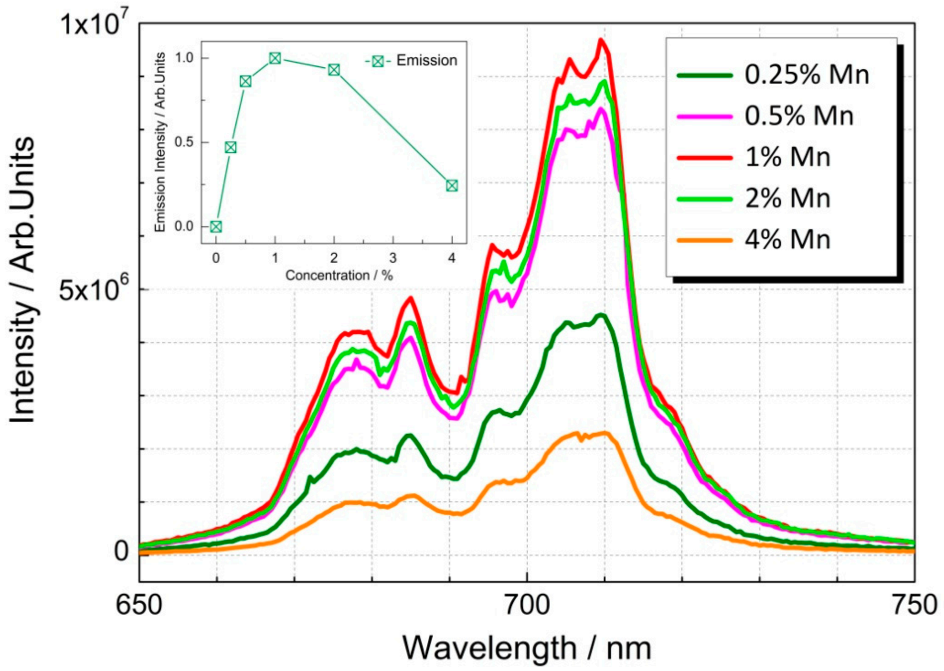

Figure S9. Emission spectra of La_2MgGeO_6:x%Mn^{4+} samples with different concentrations of Mn^{4+} (x = 0.25, 0.5, 1, 2 and 4, respectively). Emission spectra were measured under the same excitation wavelength of 309 nm. The inset illustrates the total emission intensity as a function of Mn^{4+} concentration.

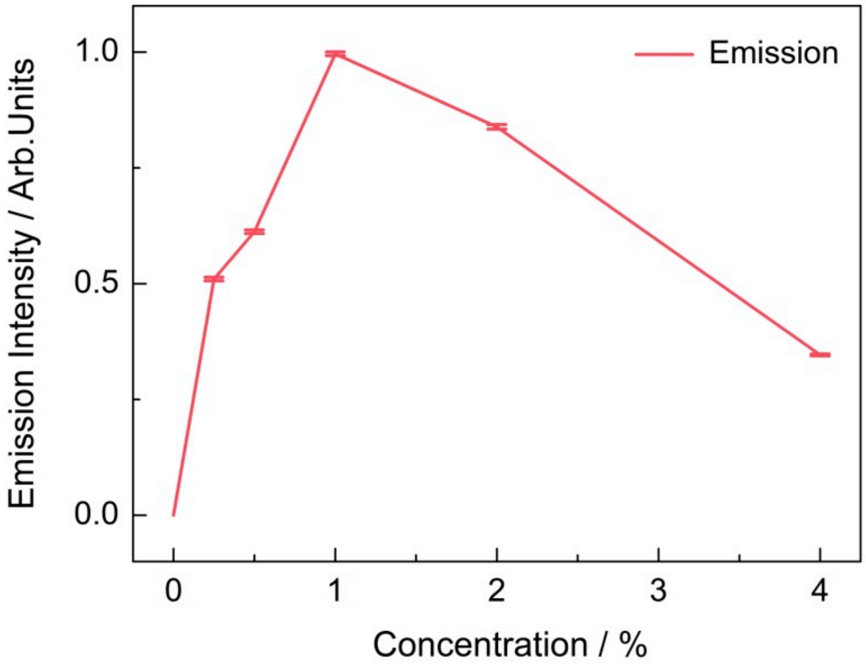

Figure S10. A comparison of emission intensity of La_2MgGeO_6:x%Mn^{4+} samples with different concentrations of Mn^{4+} (x = 0, 0.25, 0.5, 1, 2, 4) by using the integrating sphere.

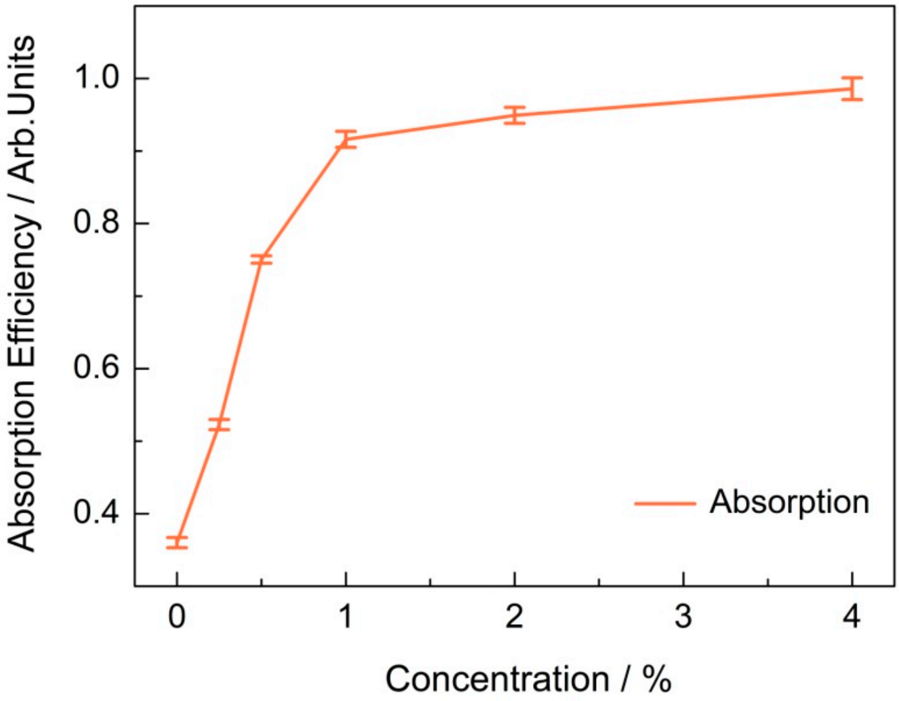

Figure S11. A comparison of absorption efficiency of La_2MgGeO_6:x%Mn^{4+} samples with different concentrations of Mn^{4+} (x = 0, 0.25, 0.5, 1, 2, 4).

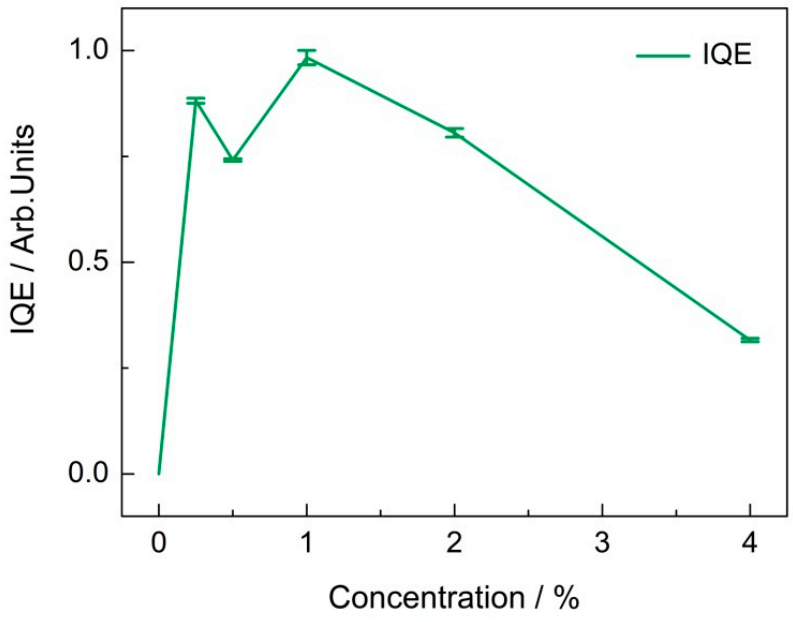

Figure S12. A comparison of internal quantum efficiency (IQE) of La_2MgGeO_6:x%Mn^{4+} samples with different concentrations of Mn^{4+} (x = 0, 0.25, 0.5, 1, 2, 4).

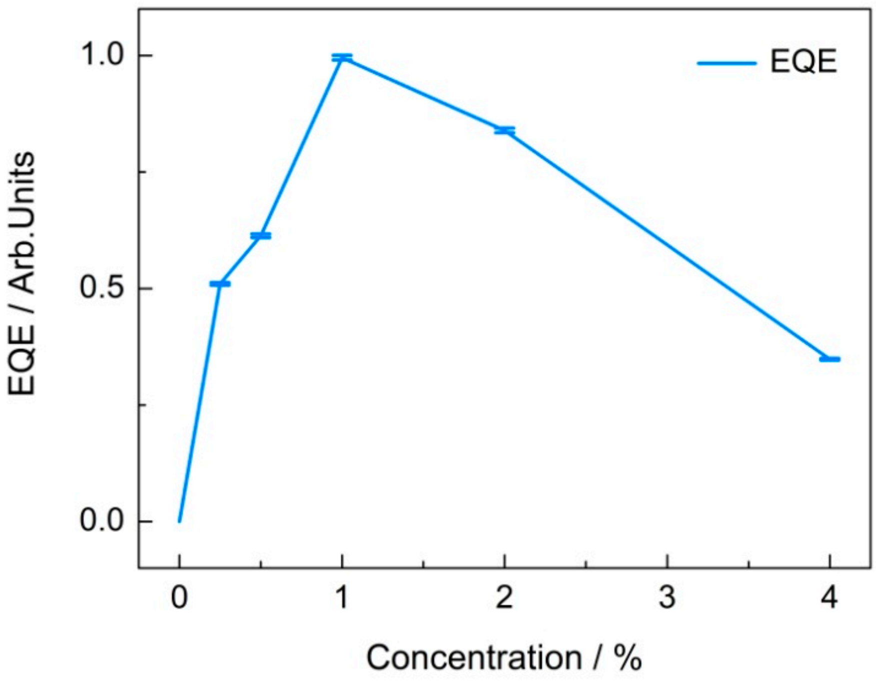

Figure S13. A comparison of external quantum efficiency (EQE) of La_2MgGeO_6:x%Mn^{4+} samples with different concentrations of Mn^{4+} (x = 0, 0.25, 0.5, 1, 2, 4).

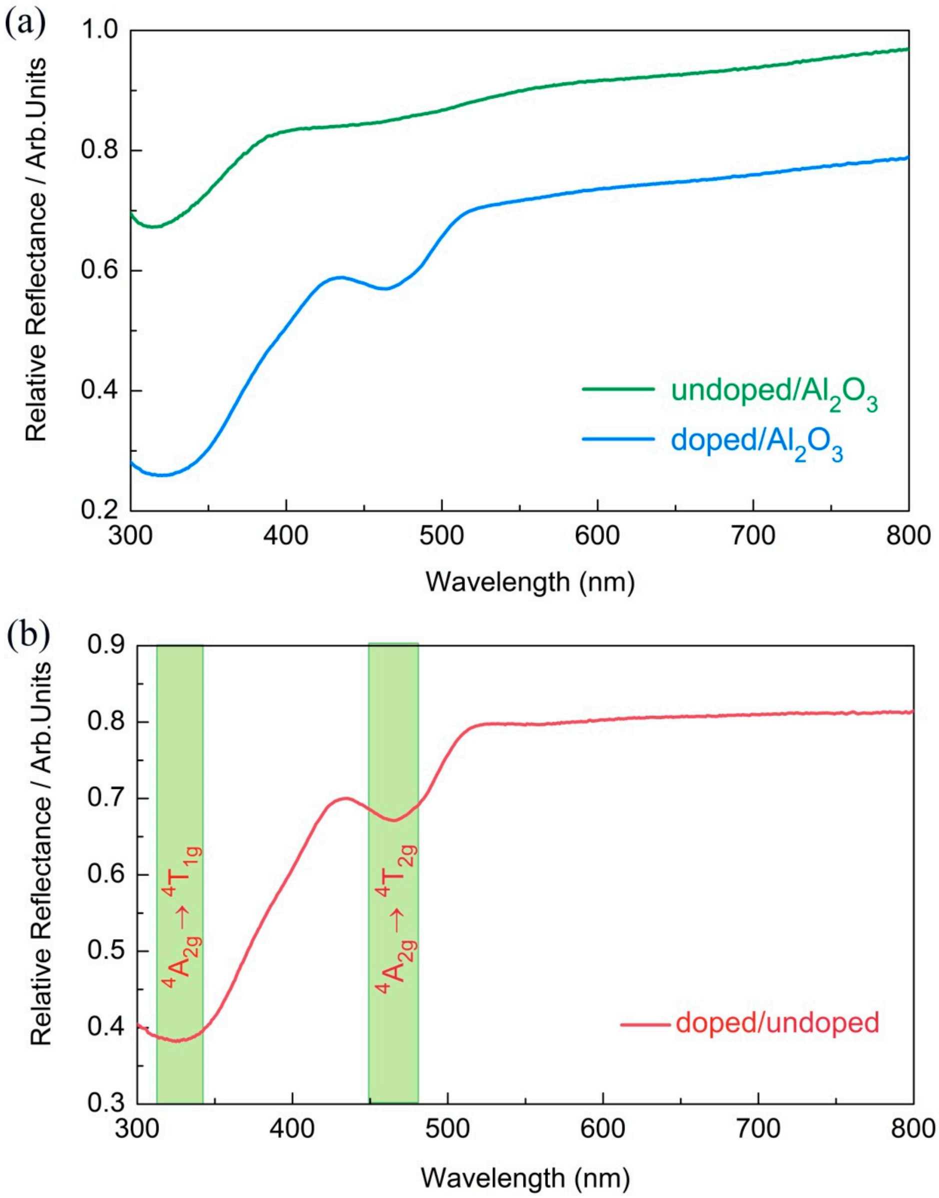

Figure S14. (**a**) Diffuse reflection spectra of the undoped La_2MgGeO_6 host, La_2MgGeO_6:1 %Mn^{4+} phosphor. (**b**) The ratio of the reflectance of 1% Mn doped La_2MgGeO_6 phosphor to the undoped La_2MgGeO_6 host.

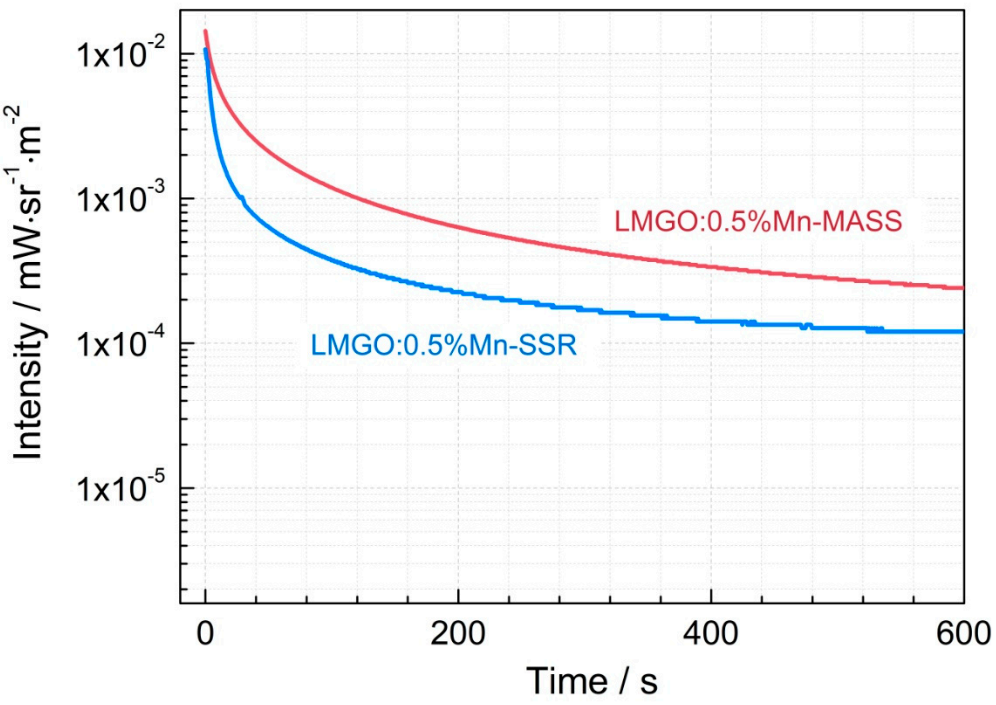

Figure S15. A comparison of the persistent luminescence decay profiles of La_2MgGeO_6:0.5%Mn^{4+} samples prepared by MASS and SSR method. The samples irradiated during 5-min prior to the decay measurement.

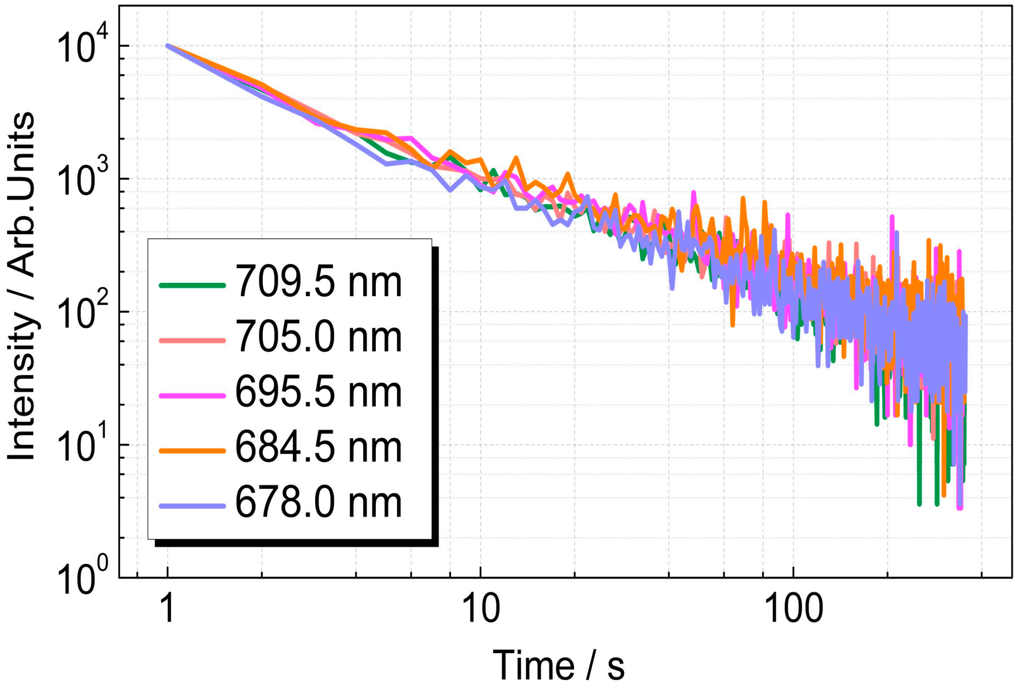

Figure S16. The emission wavelength dependent afterglow decay profiles of La_2MgGeO_6:0.5%Mn^{4+}. The afterglow decay curves were recorded monitoring at 678, 684.5, 695.5, 705 and 709.5 nm, respectively.
